# Supplementary material for: MbICE1 Confers Drought and Cold Tolerance through Up-Regulating Antioxidant Capacity and Stress-Resistant Genes in Arabidopsis thaliana
Source: Int J Mol Sci. 2022 Dec 16;23(24):16072. doi: 10.3390/ijms232416072 (PMC9783906; doi:10.3390/ijms232416072)
Supplement: Supplementary file 1 [file ijms-23-16072-s001.zip › ijms-2102631-supplementary.pdf]

```
1    ATGCTGCCAAGGCTGAACGGTGGTGTTTGGATGGACGACAGGGAGGACAGAGACTCTGTTTCCTGGACTAGAACCTCCGCCACCGCCACCCCCGGCAACACC
1     M  L  P  R  L  N  G  G  V  W  M  D  D  R  E  D  R  D  S  V  S  W  T  R  T  S  A  T  A  T  P  G  N  T

103  GAGAACAAGGACGAGATGGGTTCTTCTCTGTCTACCTTCAAGTCGATGCTGGAGGTTGAAGACGACTGGTACATGGCGGCGAATAACAGCATCCAGGGCCAT
35    E  N  K  D  E  M  G  S  S  L  S  T  F  K  S  M  L  E  V  E  D  D  W  Y  M  A  A  N  N  S  I  Q  G  H

205  TCAGATGTCGGAGACATCAGCTTCTCGCCGAGCTTTGCCGACCCAGAAAGCTTGCTGCTCAACCCGGTGGACTCTTCCTCCTCCTGCTCACCGTCGTCGTCG
69    S  D  V  G  D  I  S  F  S  P  S  F  A  D  P  E  S  L  L  L  N  P  V  D  S  S  S  S  C  S  P  S  S  S

307  GTTTTCAACAATCTCGACCCGAATCAGGTTCATTACTACATGCCTCAAAATCCCAACTTGTCTTCGCTGCTTAATGTTGTTCCTAACAACCCTTTGGATCAC
103   V  F  N  N  L  D  P  N  Q  V  H  Y  Y  M  P  Q  N  P  N  L  S  S  L  L  N  V  V  P  N  N  P  L  D  H

409  GGCTTCGATTTGGGCTGTGACATTGGGTTTCTTGACACTCAAGCTTCGGGTGGCGGTTTGAGTATGATGAACAGGGGAGGTGGGGTTTTATCTGGATTCAAT
137   G  F  D  L  G  C  D  I  G  F  L  D  T  Q  A  S  G  G  G  L  S  M  M  N  R  G  G  G  V  L  S  G  F  N

511  GATTTAAGCTCGAATAGCCAGGTGAATGCTCAGAATTTGGGTTCTAATTTACAGTTTTCGACTACCCGCATGCCTCAAGCTCTTGAAAACAGTTCAAATTTT
171   D  L  S  S  N  S  Q  V  N  A  Q  N  L  G  S  N  L  Q  F  S  T  T  R  M  P  Q  A  L  E  N  S  S  N  F

613  TCGGGTTTTCGAGGTGTGGATGGTGGTTCGGCAAATGCTTTGTTTCCCAATAGGCCTAAGTTACTGAGACCACTTGAATCCTTCCCTTCTGTGGGAGTCCAG
205   S  G  F  R  G  V  D  G  G  S  A  N  A  L  F  P  N  R  P  K  L  L  R  P  L  E  S  F  P  S  V  G  V  Q

715  CCAACTCTTTTCCAGAAAAGAGCAGCGCTTCGGAAAAACTTAGGCGATGGTGGAGGGAATTTGGGTGTTTTGGGCTCACAAGGGGGCCTGGTTTTGAATGAA
239   P  T  L  F  Q  K  R  A  A  L  R  K  N  L  G  D  G  G  G  N  L  G  V  L  G  S  Q  G  G  L  V  L  N  E

817  GGGGATGAGAGGAAGAGGGAGATGGGTGTGCAGAATGAGAAGAAGAGGAAAATGAGCGGCGGAGATGATGTGGATGATCTGAGTTTCGATGGTTCGGGTTTG
273   G  D  E  R  K  R  E  M  G  V  Q  N  E  K  K  R  K  M  S  G  G  D  D  V  D  D  L  S  F  D  G  S  G  L

919  AACTATGATTCGGATGAGTTTACTGAGAACACTAAGGTTGATGACGGTGCCAAGAATGGTGGGAACAGCTCGAATGCAAATAGCACTGTTACCGGTGGAGGA
307   N  Y  D  S  D  E  F  T  E  N  T  K  V  D  D  G  A  K  N  G  G  N  S  S  N  A  N  S  T  V  T  G  G  G

1021 GGGGGTCATAAGGGGAAGAAGAAAGGGTTGCCTGCCAAGAATCTGATGGCTGAGAGGCGCCGCCGGAAGAAGCTCAATGATAGGCTGTACATGCTGAGATCC
341   G  G  H  K  G  K  K  K  G  L  P  A  K  N  L  M  A  E  R  R  R  R  K  K  L  N  D  R  L  Y  M  L  R  S

1123 GTTGTTCCGAAGATTAGCAAAATGGACAGGGCCTCAATCCTTGGGGATGCAATTGAGTACTTGAAGGAACTTCTGCAGAGGATCAACAACCTCCACAATGAA
375   V  V  P  K  I  S  K  M  D  R  A  S  I  L  G  D  A  I  E  Y  L  K  E  L  L  Q  R  I  N  N  L  H  N  E

1225 CTGGAGTCAATCCCTTCTGGATCTGCATTGACACCTACCGGAAATACTTTCCACCCTTTGACACCCACTCCGGCTACTCTGACAAATCGTATCAAGGAAGAA
409   L  E  S  I  P  S  G  S  A  L  T  P  T  G  N  T  F  H  P  L  T  P  T  P  A  T  L  T  N  R  I  K  E  E

1327 CTTTGCCCCAGCTCATTACCTAGCCCAAATGGCCAAGCTGCAAGGGTTGAAGTTAGGCTGCGAGAAGGACGAACAGTTAACATCCACATGTTCTGCGGCCGA
443   L  C  P  S  S  L  P  S  P  N  G  Q  A  A  R  V  E  V  R  L  R  E  G  R  T  V  N  I  H  M  F  C  G  R

1429 AGGCCAGGTCTCTTGCTCTCAACCATGAGAACTTTGGATAACCTTGGGTTAGACATCCAGCAAGCTGTCATCAGCTGTTTCAATGGTTTTGCTATGGATGTC
477   R  P  G  L  L  L  S  T  M  R  T  L  D  N  L  G  L  D  I  Q  Q  A  V  I  S  C  F  N  G  F  A  M  D  V

1531 TTCCGTGCCGAGCAATGCAAGGAAGGCCAAGACGTCCATCCGGATCAGATAAAGGCGGTACTTCTGGATTCGATCGGGTTCCATGGCATGATGTAG
511   F  R  A  E  Q  C  K  E  G  Q  D  V  H  P  D  Q  I  K  A  V  L  L  D  S  I  G  F  H  G  M  M  *
```

Figure S1. The amino acid sequence of MbICE1. The part underlined in black represents the bHLH conservative domain.

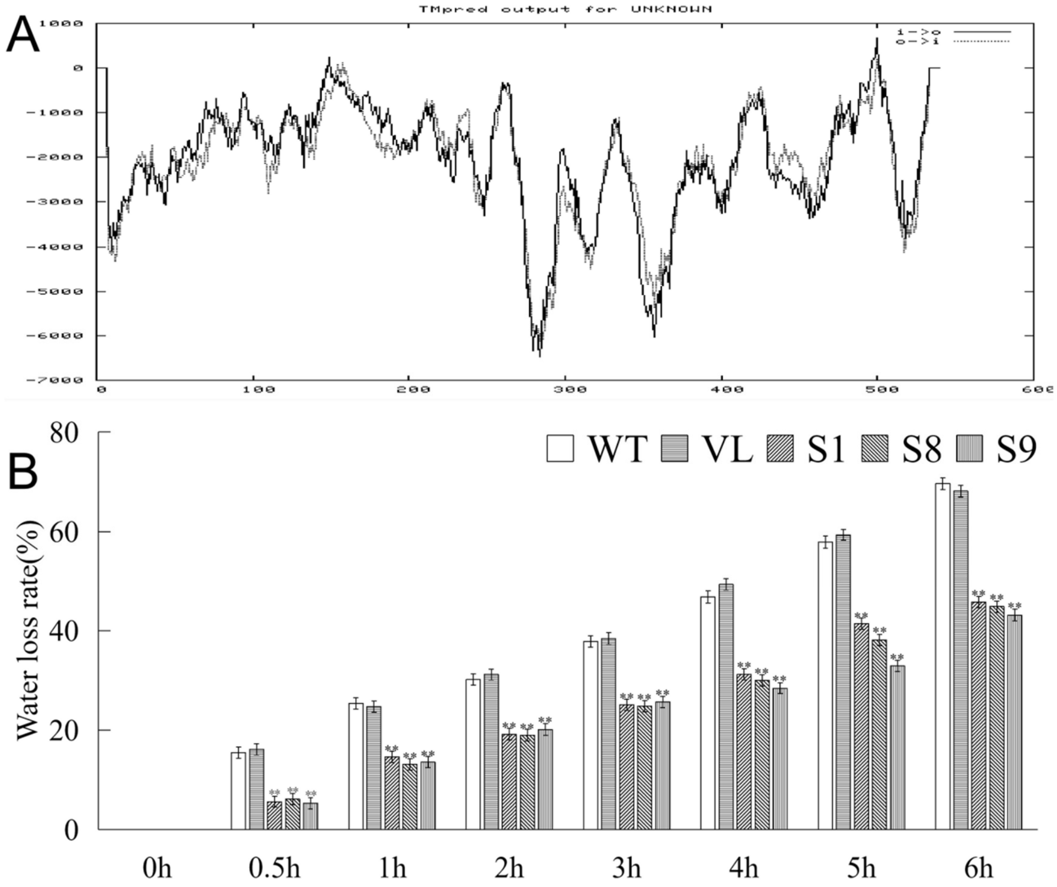

Figure S2. The transmembrane structure of MbICE1 protein and leaf water loss rate. (**A**) transmembrane structure; (**B**) water loss rate of WT and overexpression-*MbICE1 Arabidopsis* leaves at 25 ^{o}C. The value is the average of three repeats, and the standard deviation is represented by the error bar. The asterisk indicates the difference compared to WT (** $p \leq 0.01$).
